# How I do it: minimally invasive resection of a sub-ependymoma of the fourth ventricle

**DOI:** 10.1007/s00701-020-04601-5

**Published:** 2020-10-13

**Authors:** Marco V. Corniola, Torstein R. Meling

**Affiliations:** Department of Clinical Neurosciences, Division of Neurosurgery, Geneva University Hospitals, 4, Rue Gabrielle Perret Gentil, 1205 Geneve, Switzerland

**Keywords:** Minimally invasive surgery, Sub-occipital approach, Micro-surgery, Sub-ependymoma

## Abstract

**Background:**

A 54-year-old female was referred to our clinic with a lesion of the lower fourth ventricle extending to the median aperture. Here, we report the use a minimally invasive sub-occipital approach (MISA) as a safe and effective surgical management.

**Method:**

We performed a MISA using a short midline incision and a 1-cm sub-occipital craniectomy. Dissection of the lesion was performed, and “en bloc” resection could be achieved. The lesion was confirmed to be a grade I sub-ependymoma.

**Conclusion:**

MISA can be safely used when confronted to a lesion of the lower fourth ventricle.

**Electronic supplementary material:**

The online version of this article (10.1007/s00701-020-04601-5) contains supplementary material, which is available to authorized users.

## Relevant surgical anatomy

**Fig. 1:**
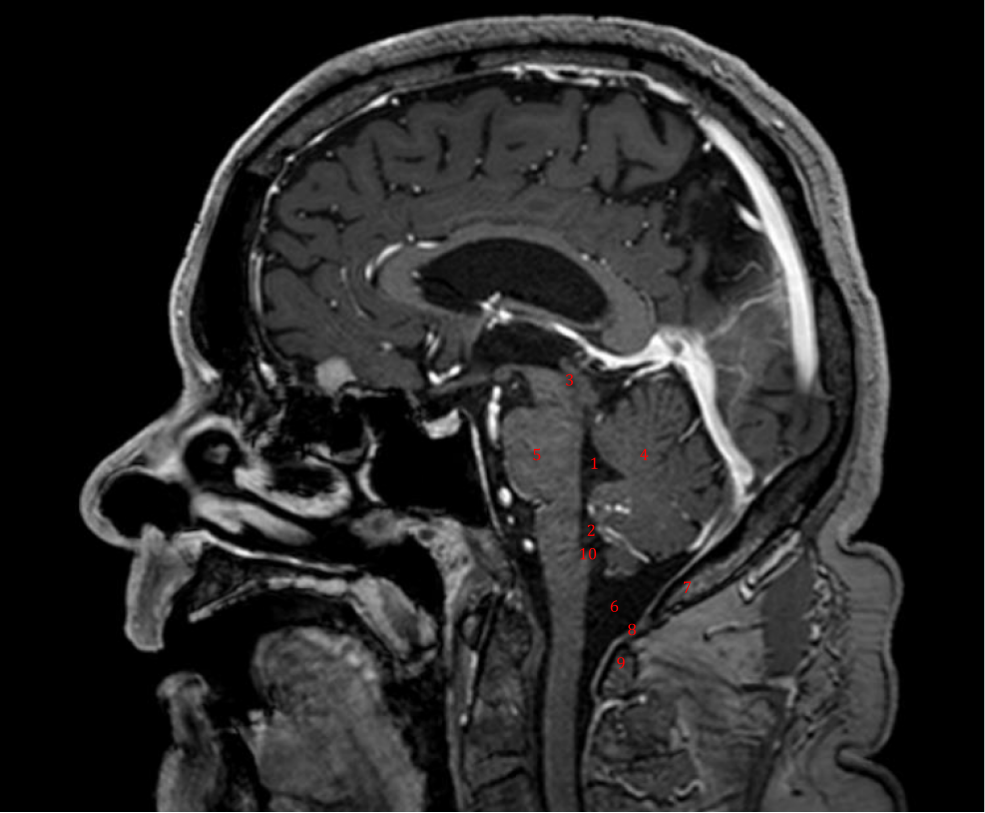
Relevant anatomy of the 4th ventricle and its surroundings. 1, fourth ventricle; 2, foramen Magendie (median aperture); 3, aqueductus sylvii; 4, cerebellum (arbor vitae); 5, pons; 6, cisterna magna; 7, planum occipital ossis occipitalis; 8, atlanto-occipital membrane; 9, posterior arch of C1; 10, obex

**Fig. 2:**
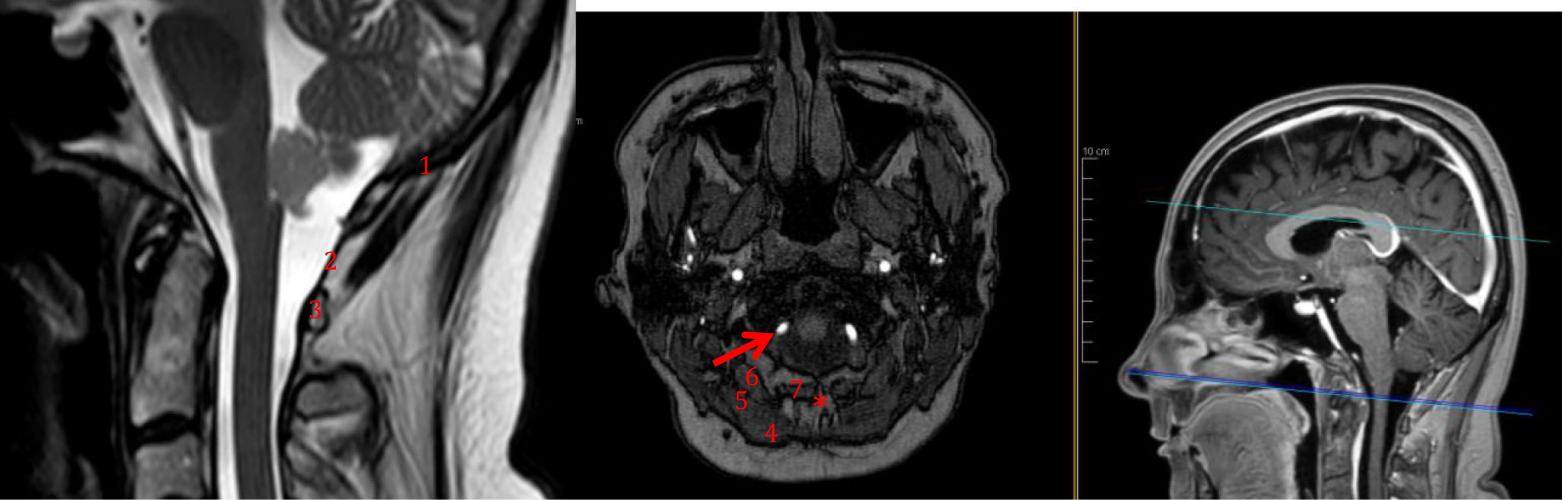
Relevant surgical anatomy of the minimally invasive sub-occipital approach. 1, lower aspect of the occipital bone; 2, atlanto-occipital membrane; 3, posterior arch of C1; 4, M. trapezius; 5, M. semispinalis capitis; 6, M. rectus capitis posterior major; 7, M. rectus capitis posterior minor; asterisk, nuchal ligament (avascular plane). Red arrow, extradural segment of the vertebral artery (V3)

The anatomy of the fourth ventricle and its surroundings is shown in Fig. 1. The specific surgical anatomy is shown in Fig. 2. The midline skin incision starts 2 to 3 cm below the external occipital protuberance and should extend circa 4 cm inferiorly. Attention should be paid to stay on the midline, i.e., into the nuchal ligament, since it is an avascular plane. Image intensifier control or navigation may be used to avoid unnecessary detachment of the posterior spinal muscles [1–6].

The superficial muscle layers consist of the trapezius (superficially) and splenius capitis (deep) muscles. The intermediate muscle layer consists of semispinalis capitis muscle; the deep muscle layer consists of rectus capitis major (lateral) and minor (medial) muscles.

**Fig. 3:**
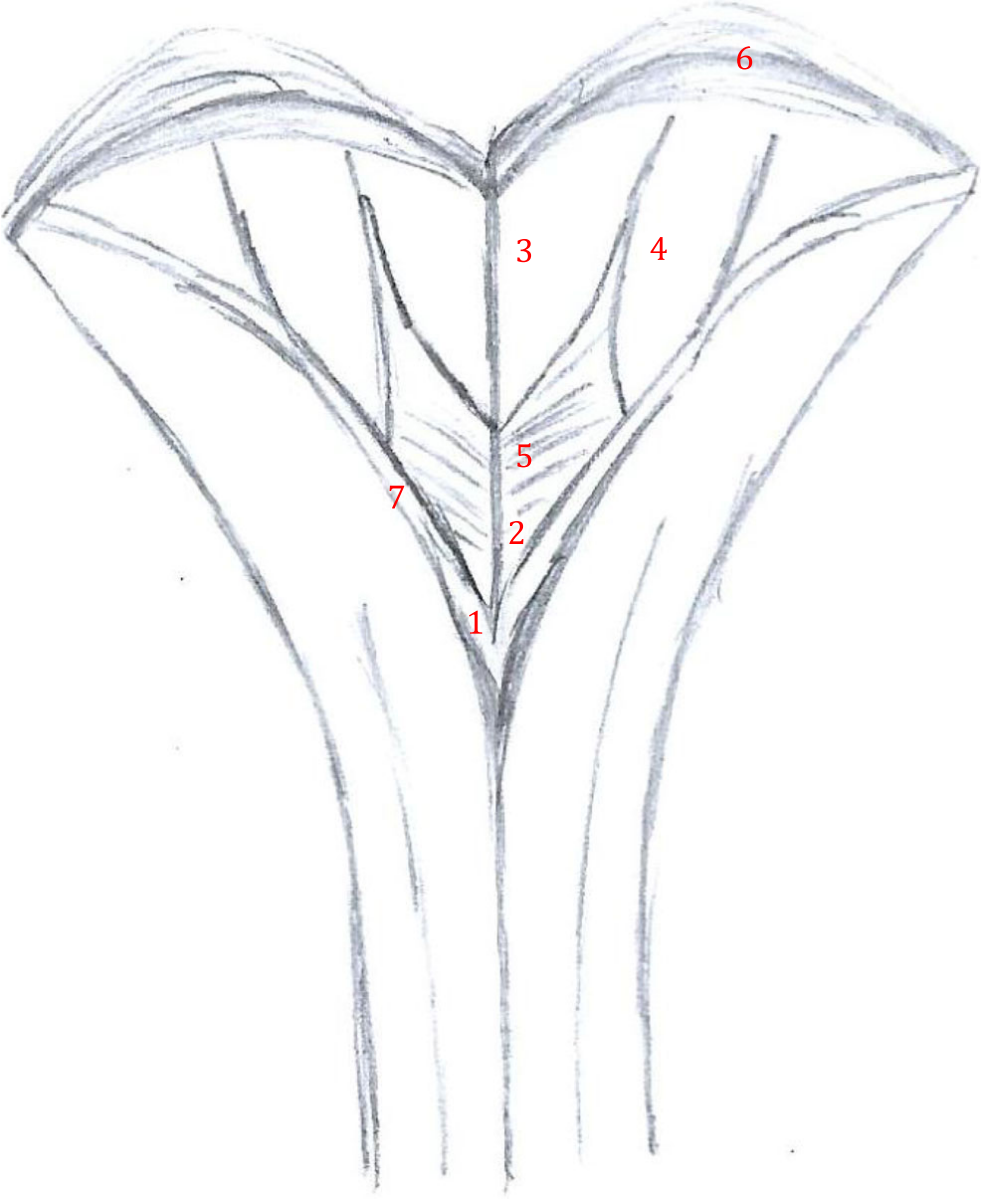
Surgical anatomy of the inferior aspect of the fourth ventricle. 1, obex; 2, area postrema; 3, trigonum nervi hypoglossi; 4, area acustica; 5, trigonum nervi vagi; 6, striae medullares; 7, taenia of the fourth ventricle

The vertebral artery turns around the lateral mass of the atlas from lateral to medial and enters into the foramen magnum through the atlanto-occipital membrane. The midline of the atlanto-occipital membrane has to be identified before the foramen magnum is carefully drilled. After opening the dura, the cisterna magna is seen. The foramen Magendie and obex are identified, in between the two cerebellar tonsils. The distal floor of the fourth ventricle may be seen (Fig. 3).

## Description of the technique

A park-bench positioning was used. The head was flexed and slightly rotated towards the floor, using a Mayfield head clamp. To avoid shifting during the surgical approach, the surgeon has to stay on the *linea nuchalis*: to facilitate orientation, the surgeon can mark the midline before turning the patient head, at the very beginning of the positioning. The head is rotated to maximize exposition and improve ergonomics. The skin was prepped and draped. Neuromonitoring bilaterally of cranial nerves IX to XII, as well as MEP and SSEP, was performed. A midline, linear incision of 4 cm was performed below the inion. The subcutaneous tissue was incised using the monopolar. Attention was paid to stay on the midline, to avoid unnecessary bleeding.

The occipital bone above the foramen magnum and the posterior atlanto-occipital membrane were exposed. Using a 5-mm sharp drill, a small craniectomy of circa 10 by 10 mm was performed. Careful hemostasis using bone wax was done. The atlanto-occipital membrane was then incised.

The dura was carefully exposed and opened in a linear fashion to facilitate a rapid and watertight closure. The dura was suspended unilaterally and the cisterna magna opened. Subsequent CSF drainage was observed. The inferior aspect of the lesion was visible in the foramen of Magendie. A thorough inspection of the inferior surface of the tumor and its surroundings was undertaken using a micro-dissector.

The tumor was slowly detached from the floor of the ventricle, with the aid of ultra-fine-tipped Zhora® forceps. Further dissection using the natural plane between the tumor and the underlying parenchyma was performed using micro-dissectors. Whenever necessary, a micro-blade knife was used to detach the tumor from the floor of the fourth ventricle. Eventually, *en bloc* resection was achieved.

Cottonoids and gentle compression were used to control a venous bleeding, avoiding direct coagulation. After careful hemostasis, a water-tight closure of the dura was achieved, using a PDS® 5-0 running suture.

**Fig. 4:**
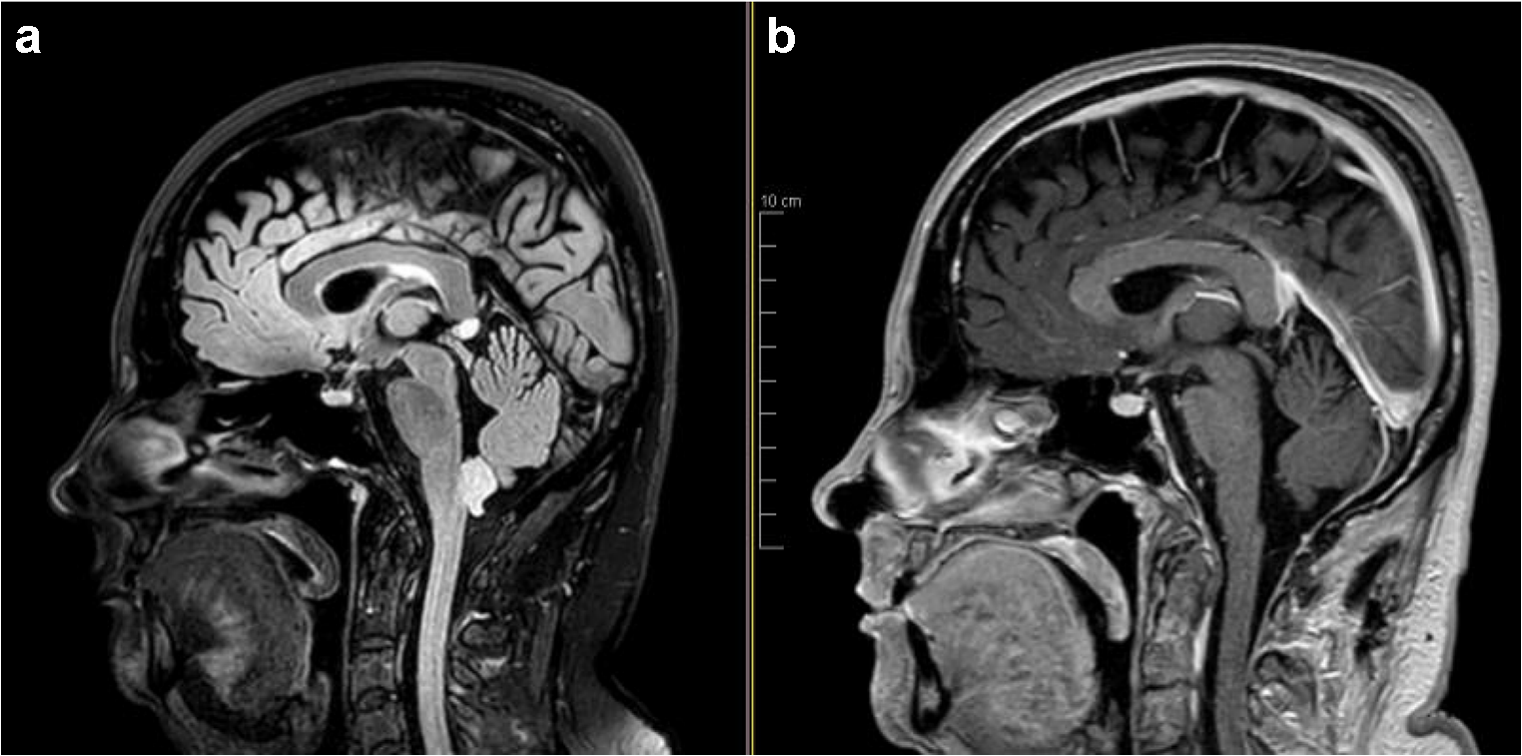
Pre- and postoperative Magnetic Resonance Imaging (**a** T2 flair; **b** T1 gadolinium), showing the complete resection of the lesion

Careful soft tissue closure was achieved using a running Monocryl® 3-0 in the muscle fascia and subcutaneous planes and a running Prolene® 4-0 for the skin closure. The postoperative MRI showed no residual tumor, with minimal stigma of the surgery (Fig. 4). The postoperative histo-pathology confirmed the diagnosis of WHO grade I sub-ependymoma.

The patient had no neurological deficits and an uneventful recovery.

## Indications

Minimally invasive sub-occipital approach (MISA) is suitable for midline lesions of the foramen magnum, as well as the lower half of the fourth ventricle. More specifically, MISA can be performed for tumoral lesions such as sub-ependymomas of the fourth ventricle of midline, infratentorial metastasis, as well as lesions of the lower vermis and cavernomas of the lower trunk, for example. The size of the craniotomy can be adjusted to the lesion.

## Limitations

Lesions with extensions to the upper half of the fourth ventricle are not candidates for MISA, since the control of the upper pole of the lesion and surroundings cannot be achieved safely. Patients with cervical arthrosis may have reduced cervical mobility, preventing proper neck flexion. Careful radiological assessment of the space between the posterior edge of the foramen magnum and the upper aspect of the posterior arch of C1 is mandatory.

## How to avoid complications

Adequate neck flexion is key to proper access to the posterior atlanto-occipital corridor (POAC). Navigation or image intensifier may be used.

During the dissection, ensure to stay on the midline to avoid bleeding. Skewing to the lateral part of the foramen magnum exposes to the risk of vertebral artery injury and related venous plexus injury.

Do not manipulate the obex and do not coagulate floor of the 4th ventricle, even if bleeding is encountered. Gentle pressure using cottonoids as well as rinsing are sufficient. The manipulation of the floor of the fourth ventricle must be avoided, since it may cause lower cranial nerves deficit (mostly swallowing disorders, eventually requiring tracheostomy).

Water-tight closure is mandatory, to prevent postoperative CSF leaks. The deep, intermediate, and superficial muscle layers, as well as the subcutaneous planes, should be closed properly.

MISA should be performed by a trained surgeon, experienced in the classic sub-occipital telovelotonsillar approach, since MISA is actually a variation of the regular telovelar approach. The minimal invasive craniectomy allows less muscle retraction (resulting in less postoperative pain) and reduces perioperative bleeding as well as the risk for postoperative cerebrospinal fluid leakage. However, working in a narrow space with limited maneuverability may result in higher risks of intra-operative bleeding or overlooking a piece of tumor, especially when performed by inexperienced surgeons.

## Specific perioperative considerations

It is important to assess the neck mobility of the patient prior to surgery. Similarly, the POAC should be accurately measured on the preoperative MRI. Patients with limited POAC may not be candidates for MISA. However, excessive flexion should be avoided, since it may impede proper venous outflow.

Propofol® is used for general anesthesia because of the postoperative nausea and vomiting prophylaxis. Postoperatively, the patient should be monitored for 24 h in a proper intermediate care unit.

## Specific information to give to the patient about surgery and potential risks

Patients should be warned about general risks of the surgery, e.g., hematoma, postoperative infection, CSF leaks, and pseudo-meningocele. Unrecognized skewing towards the lateral foramen magnum may lead to vertebral artery injury, requiring endovascular occlusion.

## Conclusion

As a variation of the regular telovelar approach, the MISA can be safely used when confronted to a lesion of the lower 4th ventricle. Careful preoperative assessment of the POAC and proper patient positioning are key to achieve complete resection with the MISA.

## Electronic supplementary material

ESM 1 (MP4 457684 kb)
